# 7, 8-Dihydroxyflavone, a TrkB receptor agonist, provides minimal protection against retinal vascular damage during oxygen-induced ischemic retinopathy

**DOI:** 10.1371/journal.pone.0260793

**Published:** 2021-12-02

**Authors:** Ismail S. Zaitoun, Yong-Seok Song, Andrew Suscha, Mohamed El Ragaby, Christine M. Sorenson, Nader Sheibani

**Affiliations:** 1 Department of Ophthalmology and Visual Sciences, University of Wisconsin School of Medicine and Public Health, Madison, WI, United States of America; 2 McPherson Eye Research Institute, University of Wisconsin School of Medicine and Public Health, Madison, WI, United States of America; 3 Department of Pediatrics, University of Wisconsin School of Medicine and Public Health, Madison, WI, United States of America; 4 Department of Cell and Regenerative Biology, University of Wisconsin School of Medicine and Public Health, Madison, WI, United States of America; 5 Department of Biomedical Engineering, University of Wisconsin School of Medicine and Public Health, Madison, WI, United States of America; Cedars-Sinai Medical Center, UNITED STATES

## Abstract

Retinopathy of prematurity (ROP) is one of the main causes of blindness in children worldwide. Brain-derived neurotrophic factor (BDNF) and its receptor, tropomyosin-related kinase B (TrkB), play critical protective roles in the development and function of neurons and vasculature. Lack of BDNF expression results in increased endothelial cell apoptosis and reduced endothelial cell-cell contact. Premature babies who develop ROP tend to have lower serum BDNF levels. BDNF expression is also significantly lower in mouse retinas following exposure to hyperoxia compared to those reared in room air. Specifically, BDNF promotes angiogenic tube formation of endothelial cells (EC), and it is considered an EC survival factor required for stabilization of intramyocardial vessels. We hypothesized that the activation of TrkB receptor protects retinal vasculature in the mice during oxygen-induced ischemic retinopathy (OIR), a model of ROP. To test this hypothesis, we treated neonatal mice with 7,8-dihydroxyflavone (DHF) (5 mg/kg body weight), a TrkB receptor agonist. We examined its potential protective effects on retinal vessel obliteration and neovascularization, two hallmarks of ROP and OIR. We found that retinas from DHF treated postnatal day 8 (P8) and P12 mice have similar levels of vessel obliteration as retinas from age-matched control mice subjected to OIR. Similarly, DHF showed no significant effect on mitigation of retinal neovascularization during OIR in P17 mice. Collectively, our studies demonstrate that the TrkB receptor agonist DHF provides no significant protective effects during OIR.

## Introduction

Retinopathy of prematurity (ROP) is a major cause of visual impairment and blindness that affects a significant proportion of children born prematurely in the USA [1]. The development of ROP is triggered by oxygen-induced disruption of the normal retinal vasculature development. ROP hallmarks comprise two pathological phases: the hyperoxia-induced vascular obliteration (ROP-Phase I) and the ensuing hypoxia-induced neovascularization (ROP-Phase II) [2]. Current medical interventions for ROP are achieved by laser ablation of the avascular part of the retina [3] and by intravitreal injection of an antibody to neutralize excess vascular endothelial growth factor (VEGF) [4–7]. However, these treatments come with drawbacks. Laser ablation may compromise the visual acuity and visual fields [8]. Anti-VEGF treatment may result in a temporal inhibition of the normal vascular development in the retina [9] and a permanent functional and structural disruption of the neuroretina [10]. In addition, anti-VEGF antibody can leak into the circulation system, which potentially can cause disruption to normal vascular and other organs development [11]. Thus, there is an urgent need for development of alternative treatments for ROP, which overcome these limitations.

Brain-derived neurotrophic factor (BDNF) activity, through binding to its receptor, tropomyosin receptor kinase B (TrkB), plays important roles in the development and function of various types of neurons [12, 13]. BDNF downregulation is implicated in many neurodegenerative and psychiatric disorders [14]. Changes in BDNF levels are also involved in diseases affecting the retina, such as diabetic retinopathy and glaucoma [15, 16]. Unfortunately, BDNF has poor diffusion [17] and pharmacokinetic characteristics, which hinders its therapeutic usage [18]. Alternatively, the small-molecule 7,8-dihydroxyflavone (DHF) has selective affinity for TrkB with strong pharmacokinetic properties including good stability and ability to cross the blood brain barrier [19]. Use of DHF as a BDNF mimic has successfully provided neuroprotective effects in some relevant neurological diseases [20–22].

BDNF/TrkB signaling pathway also plays critical roles during vascular development. Lack of BDNF expression increased endothelial cell apoptosis and reduced endothelial cell-cell contact. In contrast, overexpression of BDNF resulted in higher vascular density [23]. In vitro studies showed that BDNF promotes angiogenic tube formation of endothelial cells [24]. Along with its roles in normal vascular development, studies have reported some evidence that BDNF/TrkB signaling is involved in the regulation of vascular diseases, such as ROP. Premature babies with ROP typically have lower BDNF serum levels as compared to premature babies who do not develop ROP [25, 26], and the mRNA expression of BDNF is significantly lower in retinas from mice exposed to hyperoxia as compared with retinas from mice reared in room air [26]. In addition, BDNF mutations are associated with severe ROP [27]. Collectively, these studies suggest that BDNF/TrkB signaling could play a regulatory role in the development and progression of ROP. However, whether activation of the TrkB receptor impacts the status of ROP has not been examined. Here we assessed the utility of DHF in protecting the mouse retinal vasculature during oxygen-induced ischemic retinopathy (OIR), a mouse model of ROP. To the best of our knowledge, this is the first report that examines the in vivo effect of DHF on retinal vasculature integrity during OIR.

## Materials and methods

### Ethics statement and animals

Experiments were performed in accordance with the National Institutes of Health Guide for the Care and Use of Laboratory Animals and approved by the Institutional Animal Care and Use Committee of the University of Wisconsin School of Medicine and Public Health (IACUC assurance number: D16-00239). Adult C57BL/6J mice were obtained from the Jackson Laboratory and were bred in our mouse colony at UW-Madison. Their litters were used for the studies described below. Mice were housed and allowed *ad libitum* access to standard rodent chow and water. The day of the birth was considered postnatal day zero (P0). At desired termination time points animals were euthanatized using carbon dioxide.

### Oxygen-induced ischemic retinopathy

Oxygen-induced ischemic retinopathy (OIR) was induced in C57BL/6J mice by exposing P7 pups with their mothers to 75 ± 0.5% oxygen in an air-tight incubator for a period of 5 days. The temperature of the incubator was maintained at 23 ± 2°C, and oxygen was continuously monitored with a PROOX model 110 oxygen controller (Reming Bioinstruments Co., Redfield, NY). At the end of the 5-day period, animals were gradually introduced to room air over a period of 5–6 h before they were transferred into their regular housing area. Animals were kept under room air conditions until desired time-points were reached. Naïve C57BL/6J mice of same age were used as controls. At the end of each experiment, the animals were exposed to CO2 and death was confirmed before eyes were enucleated. For consistency and rigor, the number of pups per litter used for OIR experiments was kept small (5–7 pups). In addition, the pups’ body weight was monitored to make sure all the pups are the same size. For treatment experiments the pups were randomly divided into two groups, the treatment and control groups. Similar conditions were used for age-matched room air studies.

### Animal treatment with 7,8-dihydroxyflavone

7,8-dihydroxyflavone (DHF, Cayman Chemical, item No. 16946; CAS Number 38183-03-8) was used in these studies. The stock solution (10 mg/ml) was prepared in dimethyl sulfoxide (DMSO). The working solution was prepared in 1X PBS (3.3% DMSO in 1XPBS), as previously described by others [28, 29]. DMSO in 1X PBS without DHF was used as a vehicle. To evaluate the potential protective effect of DHF on hyperoxia-induced vessel obliteration and subsequent neovascularization, we treated neonatal C57BL/6j mice with DHF (5 mg/kg body weight) via intraperitoneal (IP) injection regimen and subjected them at P7 to 5 days of hyperoxia. Both the 5 mg/kg body weight dose and the IP route of administration are used by others [28, 29]. We analyzed the retinas from DHF and vehicle (control) treated mice for key ROP characteristics that occur during phase I (vascular obliteration) and phase II (intraretinal revascularization and epiretinal neovascularization). To study vascular obliteration at OIR P12 and OIR P8, animals were treated from P6 to P11, or from P6 to P7, respectively. To study Neovascularization and revascularization at OIR P17, animals were treated from P6 to P16 or from P12 to P16. To study the disappearance of neovascular tufts and vascular obliterated area at OIR P24, animals were treated from P6 to P23. Whenever the time of injection occurred during exposure to hyperoxia (OIR P7 to OIR P12), animals were taken out of the oxygen chamber to room-air and all of them injected with DHF or vehicle within 5 minutes and then returned to the chamber along with their mother. Once retuned to the chamber after each daily injection the oxygen level was 50% and reached to 75% within approximately 20 minutes of closing the chamber door.

### Retinal wholemount vasculature staining and quantification of vessel obliteration and neovascularization

Preparation of wholemount retina for assessing vessel obliteration and neovascularization at different time points was done as we described previously [30–32]. Briefly, the collected eyeballs were fixed (2–3 hours) in 4% paraformaldehyde then washed in PBS (3 times) before placed in methanol and kept at -20°C until used. The day of wholemount retinal immunofluorescence staining, eyeballs were rehydrated in PBS for 1 h at room temperature on the shaker, retinas were dissected in PBS, and incubated in blocking solution (1% BSA, 0.3% triton X-100 and 0.05% sodium azide in PBS) for 1 h on the shaker. Retinas were then incubated in rabbit anti-collagen IV antibody (AB756P; EMD Millipore, Burlington, MA, USA) (diluted 1/250 in the blocking buffer, fresh antibody was added daily) for 3–5 days at room temperature on the shaker. Following incubation, retinas were washed in PBS (3 times, 10 min each) before their incubation with donkey anti-rabbit secondary antibody (711-165-152; Jackson ImmunoResearch Labs, West Grove, PA, USA; 1:500 dilution prepared in blocking buffer) for 5 h at room temperature on the shaker. In some instances, retinas were co-labelled with Isolectin B4 to help in identification of the area of vessel obliteration. Before mounting, retinas were cleaned from debris (membranes/vitreous traces) labeled with rabbit-anti-collagen IV-Cy3 using a fluorescence stereomicroscope. Retinas were then mounted, and images were captured in digital format using EVOS Fluorescence Microscope. Using ImageJ, the central retinal vessel obliterated area was demarcated manually and quantified as a percentage of the whole retina area, from the captured digital images in masked fashion. Quantification of epiretinal neovascularization was performed as previously described by Stahl et al. [33]. Measurements of vessel obliteration and neovascularization are regularly performed by our group [30–32].

### RNA isolation, cDNA preparation, and quantitative PCR

RNA isolation, cDNA preparation and quantitative PCR (qPCR) were performed as we previously described [31]. Briefly, two freshly collected retinas from the same animal were snap frozen in liquid nitrogen and stored in -80°C until used. The day of RNA extraction, tubes with 2 retinas were removed from the freezer and retinas were homogenized in 500 μL Trizol solution (ThermoFisher). RNA was isolated using an established method of Trizol/Chloroform and RNeasy column (Qiagen, Valencia, CA, USA). Two retinas typically yield 5–7 μg total RNA. Total RNA (1 μg) was used to generate the complimentary deoxyribonucleic acid (cDNA) using Sprint RT Complete-Double PrePrimed kit (Clontech; Mountain View, CA) according to the manufacturer’s instructions. For each qPCR reaction, 1 μl of cDNA (dilution 1:10; contains cDNA generated from 5 nanogram RNA) was used as template on Mastercycler Realplex (Eppendorf; Hauppauge, NY) using the SYBR qPCR Premix (Clontech) with the following specific primers: Bdnf_F, AGCCGCAAAGAAGTTCCA; Bdnf_R, GCAACC GAAGTATGAAATAACCA; TrkB_F, ACGAGTATGGGAAGGATG; TrkB_R, TTCAGGG TAATTTGGGTTTG; TrkB-Variant1_F, GATGTCCCGAGATGTGTA; TrkB-Variant1_R, GGTGGTGAATTTCCTGTA; TrkB-Variant2_F, CATCTGTGGTGGGATTCT; TrkB-Variant2-R, CCAGTGGGATCTTATGAAAC. Expression of each transcript was assessed in triplicate reactions. Relative expression of targeted transcript was normalized to the housekeeping gene RpL13A using the quantitative 2^−ΔΔCT^ method [34].

### Western blot analysis

Room-air and OIR mice (P17 or P18) were injected intraperitoneally with DHF (5 mg/Kg) or control (0 hour) and retinas were harvested after 2, 4 or 6 hours. The mouse retinas (2 retinas per sample) were harvested and homogenized in 300 μL of cold lysis buffer (50 mM HEPES pH 7.5, 100 mM NaCl, 0.1 M EDTA, 1 mM CaCl2, 1 mM MgCl2, 1% Triton X-100, 1% NP-40, 0.5% deoxycholate) with proteases and phosphatase inhibitor (1861284; ThermoFisher, Pittsburg, PA). The total protein concentrations were measured using the Pierce BCA protein assay kit (23225; ThermoFisher). Thirty microgram (30 μg) of retinal lysates were separated by electrophoresis on 4–20% gradient gels (XP04202; ThermoFisher) and transferred to nitrocellulose membranes (1060001; ThermoFisher). The membranes were incubated with anti-TrkB (4603; Cell signaling, Danvers, MA; diluted 1:1000), anti-phosphorylated (Y705)-TrkB (PA5-38077; ThermoFisher; diluted 1:500), anti-phosphorylated (Y816)-TrkB (ABN1381; MilliporeSigma, St Louis, MO; diluted 1:500) and anti-β actin (MA5-15739; ThermoFisher; diluted 1:2000) antibodies. The membranes were washed with TBST (Tris Buffered Saline; 20 mM Tris-Base, 150 mM NaCl pH 7.6 containing 0.05% Tween 20) and incubated with appropriate HRP-conjugated secondary antibodies (Jackson ImmunoResearch, West Grove, PA) as recommended by the supplier. Secondary antibodies were diluted as the following: Rabbit-HRP 1:4000 for TrkB, Rabbit-HRP 1:2500 for p-TrkB (Y705 and Y816), and Mouse-HRP 1:5000 for beta actin (mouse-HRP). Following incubation, the blots were washed with TBST and developed using enhanced chemiluminescence reagents (RPN2209; ThermoFisher). The intensities of protein bands, relative to loading control, were quantified using ImageJ software and plotted using GraphPad Prism version 8 (GraphPad Software, San Diego, CA, USA).

### Statistical analysis

Statistical analysis was performed as we previously described [30], using GraphPad Prism version 8 for Windows software (GraphPad Software, La Jolla, CA). Shapiro-Wilk test was used for assessing normal distribution of the data. One outlier data point was removed from the analysis. Differences between 2 groups for morphological studies were detected using un-paired Student T-test (two-tailed). qPCR data and Western blot data were analyzed with a One-Way ANOVA. Tukey method was used for qPCR post hoc group comparisons. Tukey method is a recommended method, that utilizes pairwise post-hoc analysis, to determine the significant differences between the means of every possible two groups of all examined groups [35]. Dunnett’s multiple comparisons test was used for Western blot data. Dunnett method is a recommended method to analyze studies that have control group(s); It is used to test two or more experimental groups against the control group [35]. Presented data are Mean ± standard deviation. P<0.05 was considered significant.

## Results

### Retinal expression of Bdnf and Ntrk2, and DHF-induced TrkB activation in room-air mice

**Fig 1:**
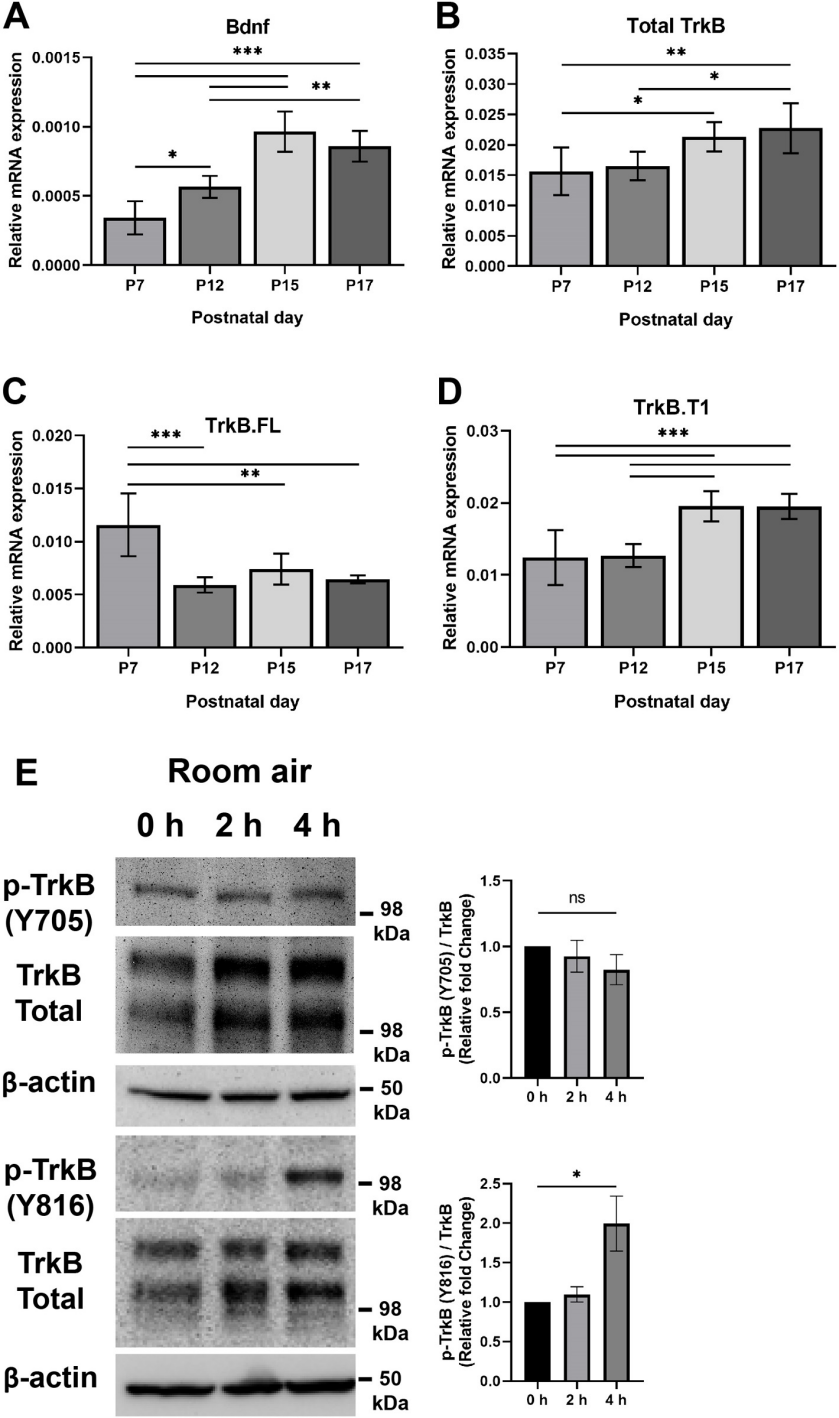
The expression of Bdnf and TrkB in the retinas from room-air mice. The mRNA expression level was assessed in retinas from P7, P12, P15 and P17 room-air mice. **Panel A**, expression of Bdnf showed significant upregulation with age and reached a peak at P15; Bdnf level at P17 showed some downregulation but was not significant when compared to retinas from P15 mice. Actual p-values: P7 vs. P12: *P = 0.0161; P7 vs. P15: ***P< 0.0001; P7 vs. P17: ***P< 0.0001; P12 vs. P15: ***P< 0.0001; P12 vs. P17: **P = 0.0015; P15 vs. P17: P = 0.4149. **Panel B**, expression of total TrkB in retinas showed an upregulation trend with age. Its level at P7 was significantly different from those at P15 and P17 time points and its level at P12 was significantly different from P17 time point. Actual p-values: P7 vs. P12: P = 0.968; P7 vs. P15: *P = 0.0341; P7 vs. P17: **P = 0.0067; P12 vs. P15: P = 0.0857; P12 vs. P17: *P = 0.0185; P15 vs. P17: P = 0.8793. **Panel C**, expression of TrkB.FL in retinas at P7 was significantly higher than levels at all other time points examined in this study; Levels of TrkB.FL at P12, P15 and P17 were similar to each other. Actual p-values: P7 vs. P12: ***P< 0.0001; P7 vs. P15: **P = 0.002; P7 vs. P17: ***P = 0.0002; P12 vs. P15: P = 0.4379; P12 vs. P17: P = 0.9478; P15 vs. P17: P = 0.7562. **Panel D**, expression levels of TrkB.T1 in retinas at P7 and P12 time points were similar. Levels in retinas at P15 and P17 times points were similar to each other as well, but they were significantly increased compared with levels in retinas from either P7 or P12 time points. Actual p-values: P7 vs. P12: P = 0.9974; P7 vs. P15: ***P = 0.0004; P7 vs. P17: ***P = 0.0004; P12 vs. P15: ***P = 0.0006; P12 vs. P17: ***P = 0.0006; P15 vs. P17: P> 0.9999. **Panel E**, effect of DHF administration on TrkB phosphorylation in the mouse retina. Room-air mice were intraperitoneally administrated with DHF for 2 or 4 hours before retinas were collected and processed. Retinal lysates (30 μg) were analyzed by western blot analysis for phosphorylated (Y816 and Y705) TrkB and total TrkB using specific antibodies. The β-actin expression was used as a loading control. Treating P18 room-air animals with DHF resulted in a significant increase in the phosphorylation of the Y816 residue after 4 hours of treatment, P = 0.0285 but not after 2 hours of treatment, P = 0.8606. However, treating room-air animals with DHF did not result in significant changes in the phosphorylation of the Y705 residue after 2 hours of treatment, P = 0.6967 or after 4 hours of treatment, P = 0.2592. For Panel E, samples were assessed twice. Mean ± SD; significance was determined using One-Way ANOVA, *P≤ 0.05.

We assessed mRNA expression levels of Bdnf, and Ntrk2 in retinas from P7, P12, P15 and P17 mice kept at room-air (Fig 1A–1D). Expression of Bdnf showed significant upregulation with age and reached a peak at P15; Bdnf level at P17 showed some downregulation but was not significant when compared to retinas from P15 mice (Fig 1A). Ntrk2 is the gene that encodes for Bdnf receptor, TrkB. Ntrk2 expresses two major variants in the central nervous system [36]. Variant 1 (referred to as TrkB.FL) encodes the full-length TrkB protein, which includes the tyrosine kinase domain that autophosphorylates upon binding to Bdnf protein. Variant 2 (referred to as TrkB.T1) is shorter and encodes the truncated TrkB protein, which lacks the tyrosine kinase domain but still binds to Bdnf. Next, we assessed the total level of TrkB (same pair of primers exist in both variant 1 and variant 2) (Fig 1B), the TrkB.FL (Fig 1C) and TrkB.T1 (Fig 1D). Expression of total TrkB in retinas showed an upregulation trend with age. Its level at P7 was significantly different from those at P15 and P17 time points and its level at P12 was significantly different from P17 time point (Fig 1B). Expression of TrkB.FL in retinas at P7 was significantly higher than levels at all other time points examined in this study; the levels of TrkB.FL at P12, P15 and P17 were similar to each other (Fig 1C). Expression levels of TrkB.T1 in retinas at P7 and P12 time points were similar. Levels in retinas at P15 and P17 times points were similar to each other as well, but they were significantly increased as compared to the levels in retinas from either P7 or P12 time points (Fig 1D). Noteworthy, qPCR data showed that the truncated variant was more abundant than the full-length variant.

To determine whether the intraperitoneal (IP) delivery of DHF can reach the retina and activate TrkB, we treated room-air mice with DHF or vehicle and determined the retinal levels of TrkB phosphorylation by Western blot analysis. DHF treatment was shown by others to promptly increase phosphorylation of TrkB as early as 10 minutes and phosphorylation steadily decrease after 8 hours and is nearly undetectable after 16 hours in retinal ganglion cells [37]. Therefore, we assessed TrkB phosphorylation levels in retinas from room-air animals after 2 and 4 hours of injection with DHF or vehicle, which is similar to in vivo studies reported by others [19, 28, 29]. We assessed the phosphorylation level of TrkB at two different tyrosine residues: Y816 (the phospholipase Cγ1 binding site) and Y705 (the autophosphorylation site) [38, 39] using phospho-specific antibodies. Treating room-air animals with DHF resulted in a significant increase in phosphorylation of the TrkB Y816 residue after 4 hours of injection with minimal effect on the phosphorylation of Y705 residue (Fig 1E). Thus, DHF reaches the retina and result in the TrkB receptor phosphorylation after at least 4 hours under room-air conditions.

### DHF treatment during OIR Phase I showed no significant protective effect against vessel obliteration

**Fig 2:**
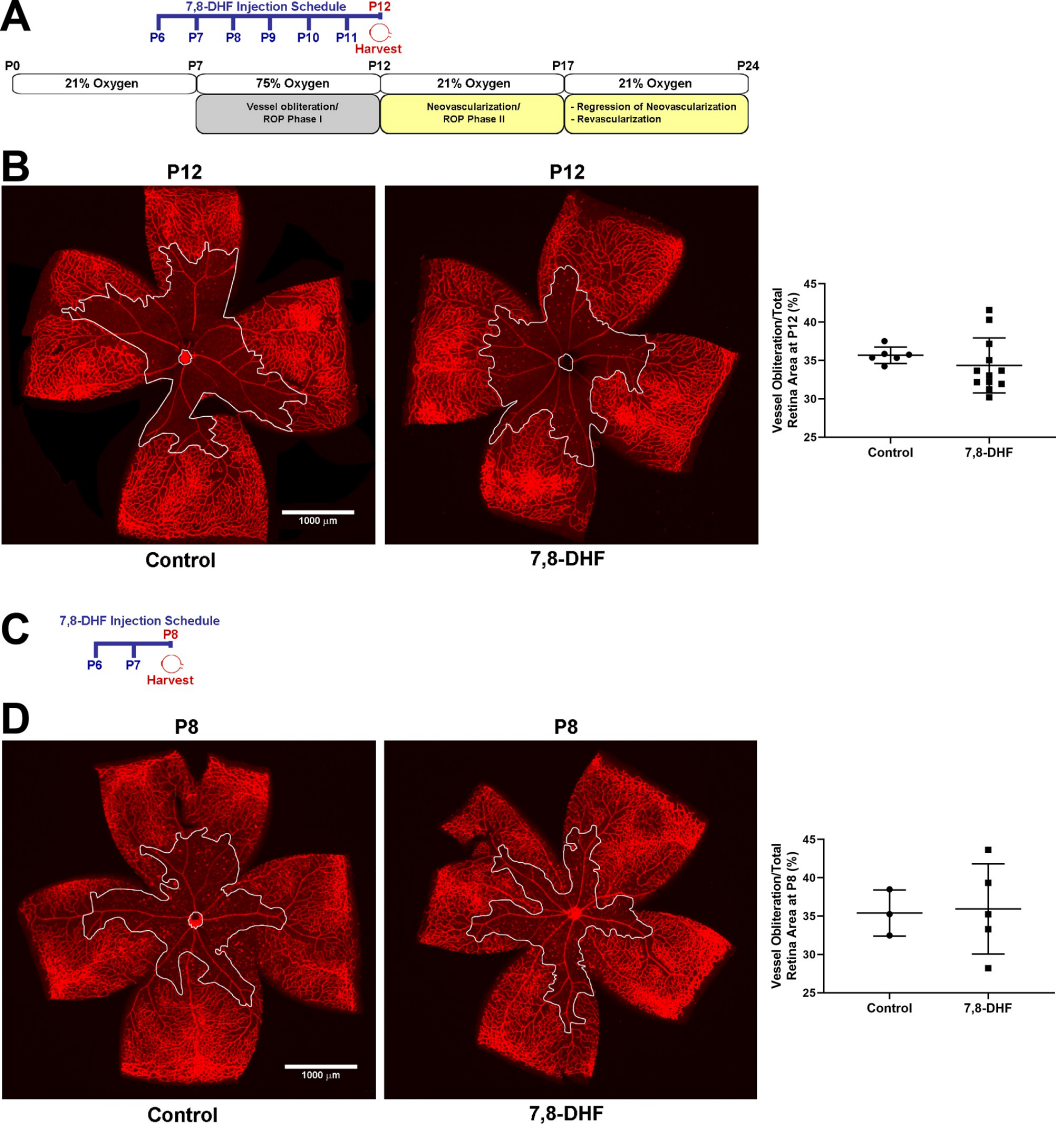
DHF does not attenuate retinal vessel obliteration at P12. **Panel A** is a schematic diagram of mouse OIR model and time points of DHF treatment. Mouse OIR model: pups are kept from birth until P7 with their nursing mother in room-air oxygen level (21% oxygen), at P7 they are exposed to 75% oxygen level for 5 days which results in vessel obliteration from the center of the retina (equivalent to ROP Phase I in human). At P12, pups are returned to room-air (21% oxygen). Exposure to 21% oxygen when the center of the retina is void of blood vessels results in relative hypoxia which causes both; 1) physiologic revascularization toward the vessel obliterated area and 2) pathologic epiretinal neovascularization that peaks at P17 (equivalent to ROP phase II in human) before it starts to regress. When OIR pups are kept in room-air (21% oxygen) until P24, obliterated area in the center of the retina gets completely revascularized and all neovascular tufts regress, again events are similar to what happens in humans with ROP. DHF was injected daily, starting P6, one day before high oxygen exposure, until P11, one day before harvest at P12. **Panel B**, Animals were exposed to 75% oxygen from P7-P12. Retinas from P12 control and DHF daily treated mice were wholemount stained with Isolectin B4 and representative images and their vessel obliterated area (demarcated in a white line) relative to the total retinal area are shown. Data are derived from 6 control and 12 treated retinas (P = 0.3933). **Panel C**, DHF treatment schedule of OIR P8. DHF was injected on P6 and P7 and eye collected on P8, one day after high oxygen exposure. Animals were exposed to 75% oxygen from P7-P8. **Panel D**, retinas from P8 control and DHF treated mice were wholemount stained with Isolectin B4 and representative images and their vessel obliterated area (demarcated in a white line) relative to the total retinal area are shown. Data are derived from 3 control and 5 treated retinas (P = 0.8901). Mean ± SD; significance was determined using unpaired t-test, *P≤0.05, **P<0.01, Scale bar = 1,000 μm.

Neonatal mice were treated daily with either DHF or vehicle solution starting from one day before exposure to hyperoxia at P7 until one day before returning to room air at P12 (treated, P6-P11) according to OIR method. Retinas from P12 mice were wholemount immunostained with Isolectin B4 for visualizing blood vessels. Vascular obliteration was quantified by measuring the avascular area in the center of the retina around the optic nerve. Fig 2A and 2B shows representative images captured from wholemount retinas of DHF and vehicle treated groups along with summary of quantitative data. Retinas from DHF and vehicle treated animals exhibited similar areas of vessel obliteration. Thus, DHF did not provide retinal vascular protection against hyperoxia during OIR Phase I.

Most of the vessel obliteration occurs within the first 24 h after exposing P7 mice to 75% oxygen [40]. To examine whether DHF treatment provides temporal protection against such early vascular damage, we treated neonatal mice at P6 and P7 with DHF or vehicle and exposed them to hyperoxia for one day (P7 to P8). Wholemount retinas from both groups showed similar vessel obliteration (Fig 2C and 2D). These results demonstrate the lack of DHF protective effects against hyperoxia-mediated vessel obliteration.

### DHF treatment during OIR Phase II showed no significant protective effect on the physiologic revascularization or pathologic neovascularization events

**Fig 3:**
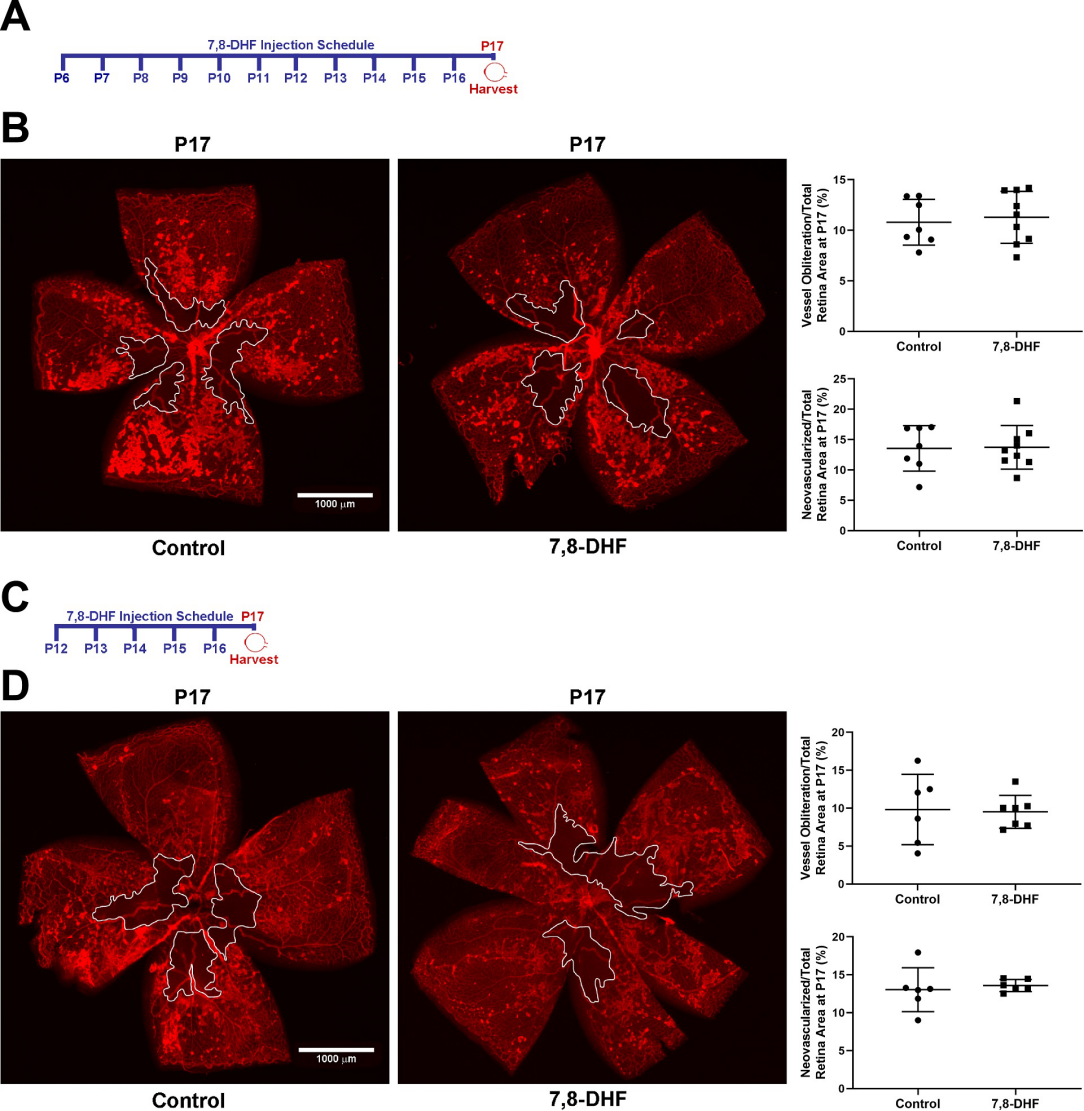
DHF does not affect physiologic revascularization or pathologic neovascularization at P17. **Panel A**, DHF treatment schedule. DHF was injected daily from P6 until P16, and eyes were collected at P17. Animals were exposed to 75% oxygen from P7-P12 then kept at room-air conditions until P17. **Panel B**, retinas from P17 control and DHF daily treated mice were wholemount stained with anti-collagen-IV and representative images and their vessel obliterated area (demarcated in a white line) relative to the total retinal area and epiretinal neovascularization are shown. Data are derived from 7 control and 9 treated retinas (vessel obliteration P = 0.7019 and neovascularization P = 0.9245). **Panel C**, DHF treatment schedule: daily from P12 until P16 and eyes collected at P17. Animals were exposed to 75% oxygen from P7-P12 then kept at room-air conditions until P17. **Panel D**, retinas from P17 control and DHF daily treated mice were wholemount stained with anti-collagen-IV and representative images and their vessel obliterated area (demarcated in a white line) relative to the total retinal area and epiretinal neovascularization are shown. Data are derived from 6 control and 7 treated retinas (vessel obliteration P = 0.8805 and neovascularization P = 0.354). Mean ± SD; significance was determined using unpaired t-test, *P≤0.05, **P<0.01. Scale bar = 1,000 μm.

To determine whether DHF can favorably affect the retinal vasculature during OIR phase II, neonatal mice were exposed to hyperoxia for 5 days (OIR Phase I) and then kept at room air for additional 5 days (OIR phase II) before retinas were immunostained with anti-collagen IV antibody and analyzed; collagen IV is a basement membrane protein that is specifically localized in the retina on the outside of blood vessels. Daily treatment with DHF or vehicle started on P6 and continued until P16, one day before collecting the eyes at P17. Fig 3A and 3B shows representative images from wholemount retinas of P17 DHF and vehicle treated mice along with the summary of quantitative data. Physiologic revascularization was quantified in wholemount retinas by calculating the avascular area relative to the total retinal area. In addition, the neovascularization was quantified by calculating total area occupied by neovascular tufts relative to total retinal area. Retinas from DHF and vehicle treated animals showed no significant differences in revascularization and neovascularization.

We next determined whether treating animals only during OIR phase II (P12-P16) would have a different outcome. Fig 3C and 3D shows representative retinal images from DHF, and vehicle treated animals. Retinas from DHF treated mice showed no significant differences in revascularization and neovascularization compared with retinas from vehicle treated animals.

**Fig 4:**
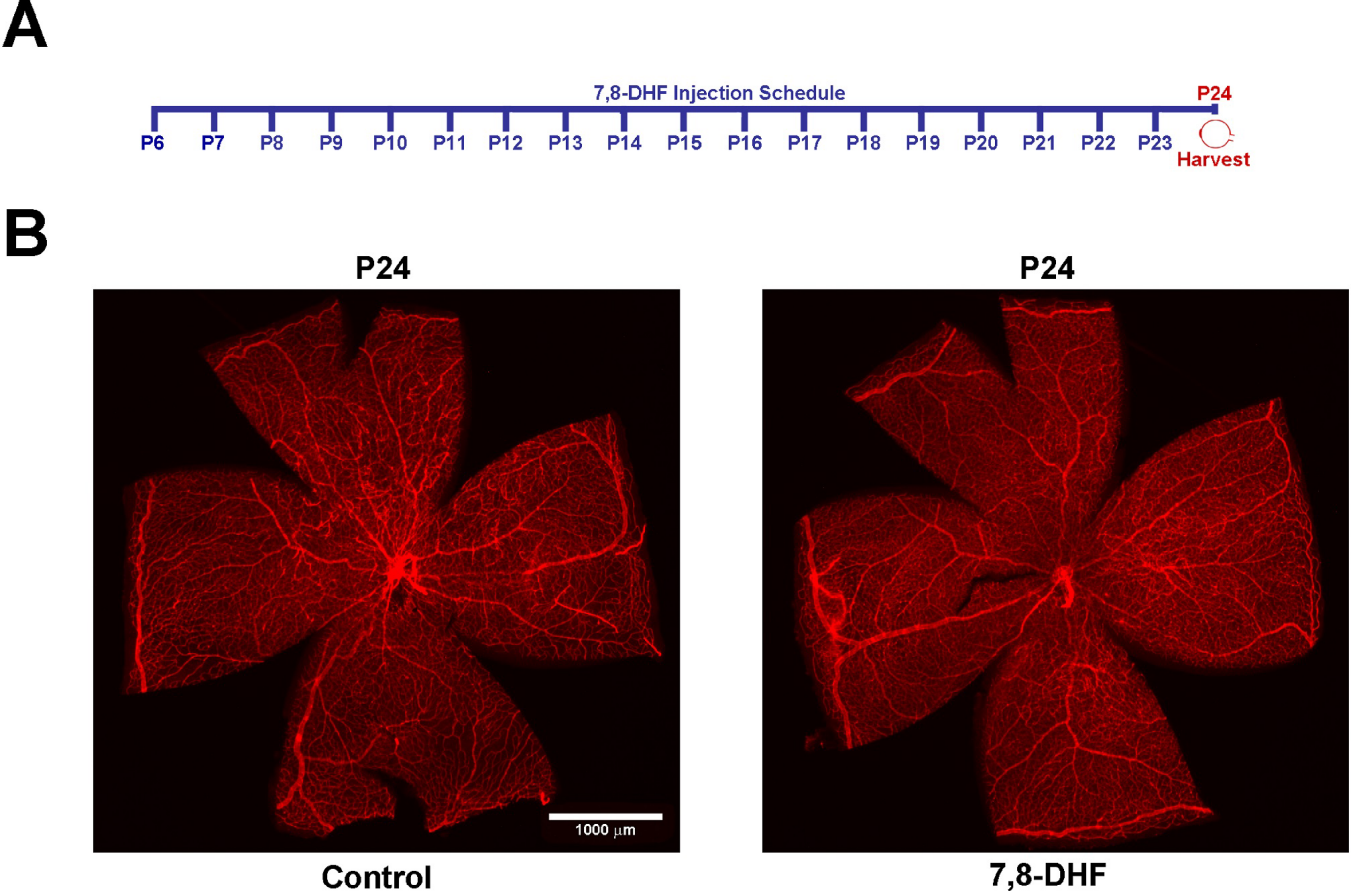
DHF does not affect the resolution of both the avascular area and neovascularization. **Panel A**, DHF treatment schedule: daily from P6 until P23 and eyes collected at P24. Animals were exposed to 75% oxygen from P7-P12 then kept at room-air conditions until P24. **Panel B**, retinas from P24 control and DHF daily treated mice were wholemount stained with anti-collagen-IV and representative images are shown. Eyes from 5 mice were used. Scale bar = 1,000 μm.

Intraretinal revascularization activity results in complete vascularization of the avascular area in the central retina by postnatal day 25 (P25). Also, all neovascularization tufts regress by P25 [40, 41]. To examine any potential effects of DHF treatment on resolution of both the avascular area and neovascularization, animals exposed to OIR were treated with DHF or vehicle from P6 to P23, and eyes were collected, and retinas were analyzed at P24. Wholemount retinas from both treatment groups showed full resolution of avascular area and neovascularization tufts (Fig 4A and 4B). Collectively, the data presented here show no significant protective effect is provided during OIR by DHF treatment.

### Retinal expression of Bdnf and Ntrk2, and DHF-induced TrkB activation in OIR mice

**Fig 5:**
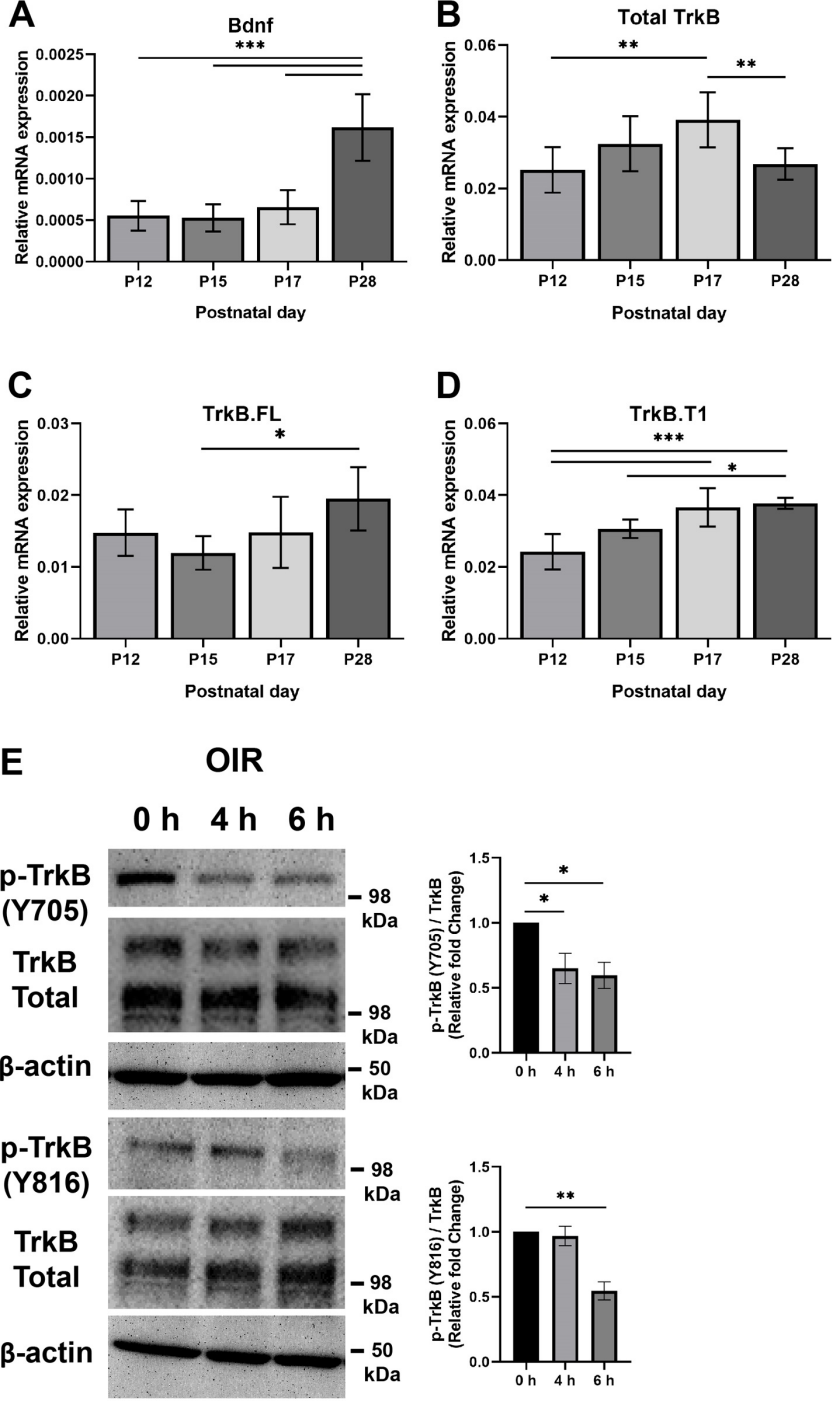
The expression of Bdnf and TrkB in the retinas from mice exposed to OIR. The mRNA expression level was assessed in retinas from OIR-P12, OIR-P15, OIR-P17 and OIR-P28 mice. **Panel A**, expression of Bdnf showed similar levels in retinas at OIR-P12, OIR-P15 and OIR-P17 time points but was significantly upregulated in retinas at OIR-P28. Actual p-values: P12 vs. P15: P = 0.998; P12 vs. P17: P = 0.8985; P12 vs. P28: ***P< 0.0001; P15 vs. P17: P = 0.82; P15 vs. P28: ***P< 0.0001; P17 vs. P28: ***P< 0.0001. **Panel B**, expression of total TrkB showed an upregulation trend with age and significantly peaked at OIR-P17 before it showed significant downregulation at OIR-P28 as compared to OIR-P17. Actual p-values: P12 vs. P15: P = 0.1644; P12 vs. P17: **P = 0.0018; P12 vs. P28: P = 0.9473; P15 vs. P17: P = 0.2954; P15 vs. P28: P = 0.3631; P17 vs. P28: **P = 0.0062. **Panel C**, expression of TrkB.FL in retinas showed similar levels in retinas at OIR-P12, OIR-P15, and OIR-P17 time points. However, its level at P28 was significantly upregulated as compared to OIR-P15. Actual p-values: P12 vs. P15: P = 0.6269; P12 vs. P17: P> 0.9999; P12 vs. P28: P = 0.2106; P15 vs. P17: P = 0.5488; P15 vs. P28: *P = 0.0181; P17 vs. P28: P = 0.1538. **Panel D**, expression levels of TrkB.T1 in retinas showed an upregulation trend with age. Its level at P28 was significantly higher than its levels at OIR-P12 and OIR-P15 time points. Also, its level at P17 was significantly higher that its level at OIR-P12. Actual p-values: P12 vs. P15: P = 0.0523; P12 vs. P17: ***P = 0.0002; P12 vs. P28: ***P< 0.0001; P15 vs. P17: P = 0.0732; P15 vs. P28: *P = 0.0264; P17 vs. P28: P = 0.9592. **Panel E**, P17 OIR mice were intraperitoneally injected with DHF for 4 and 6 hours before retinas were collected and processed. DHF treatment resulted in significant reduction in the phosphorylation of the Y705 residue after 4 hours of treatment, P = 0.0477 and after 6 hours of treatment, P = 0.0327. Similarly, phosphorylation of the Y816 residue was significantly reduced after 6 hours of treatment, P = 0.0076, but not after 4 hours of treatment, P = 0.8279. For Panel E samples were assessed twice. Mean ± SD; significance was determined using One-Way ANOVA, *P≤0.05, **P<0.01.

We assessed mRNA expression levels of Bdnf, total TrkB, TrkB.FL and TrkB.T1 in retinas from OIR-P12, OIR-P15, OIR-P17 and OIR-P28 mice (Fig 5A–5D). Expression of Bdnf showed similar levels in retinas at OIR-P12, OIR-P15 and OIR-P17 time points but was significantly upregulated in retinas at OIR-P28 (Fig 5A). Although the expression of Bdnf was increased from P12- P17 in room air mice retina, this was not the case in OIR-mice (Figs 1A and 5A). The Bdnf expression in OIR-P12 retina was comparable to room air-P12 retina, but unlike room air-P15 retina, the Bdnf expression did not increase in OIR-P15 retina. Thus, increased expression of Bdnf mRNA in OIR-P15 was mitigated during hypoxia exposure. However, the Bdnf expression was increased in OIR-P28 retina and was comparable to the levels noted in room air-P17 retina.

Expression of total TrkB showed an upregulation trend with age and significantly peaked at OIR-P17 before it showed significant downregulation at OIR-P28 as compared to OIR-P17 (Fig 5B). Expression of TrkB.FL in retinas showed similar levels at OIR-P12, OIR-P15, and OIR-P17 time points. However, its level at P28 was significantly upregulated as compared to OIR-P15 (Fig 5C). Expression levels of TrkB.T1 in retinas showed an upregulation trend with age. Its level at P28 was significantly higher than its levels at OIR-P12 and OIR-P15 time points. Also, its level at P17 was significantly higher than its level at OIR-P12 (Fig 5D). It is important to note that qPCR data showed that the truncated variant was more abundant than the full-length variant in retinas from OIR mice, which is similar to the pattern observed in retinas from room-air mice.

To determine whether DHF can activate TrkB in OIR mice, we treated animals with DHF or vehicle and determined the retinal levels of TrkB phosphorylation by Western blot analysis. We assessed TrkB phosphorylation levels in retinas from OIR animals after 4 and 6 hours of injection with DHF or vehicle; we decided to examine the level of phosphorylation 6 hours after DHF treatment instead of the 2 hours used in room-air conditions because the later time point failed to show any significant difference in phosphorylation after DHF treatment. Phosphorylation levels of TrkB at tyrosine residues Y816 and Y705 were assessed using phospho-specific antibodies. DHF treatment of OIR mice did not show a significant increase in phosphorylation level of TrkB at either residue (Fig 5E) but rather was significantly decreased by 6 hours post treatment. This may explain, at least in part, the lack of significant protection against vessel obliteration or neovascularization by DHF in retinas from mice subjected to OIR.

## Discussion

Retinal vascular development and homeostasis are strictly controlled by oxygen and nutrients needs of the neural retina. Interruption of normal oxygen level during active angiogenesis in premature babies can result in the development of ROP [2, 41]. The hallmarks of ROP are hyperoxia-mediated vessel obliteration and ischemia-mediated neovascularization. The mouse OIR is a preclinical model that recapitulates the key pathologic characteristics of ROP. Per OIR method, neonatal mice kept in room air (21% oxygen) from birth (postnatal day zero, P0) until P7. The P7 neonatal pups along with their nursing mothers are then exposed to 75% oxygen for 5 days (P7-P12) before they are returned to room air (P12-P17). During the first seven days of postnatal life, growing in room air allows the normal vascular development to take place, where the superficial vascular layer spreads from the optic nerve radially towards the periphery covering most of the retinal area. After exposure to 75% oxygen, this hyperoxic condition induces vessel obliteration to the superficial layer in the center of the mouse retina around the optic nerve (ROP Phase I). At the same time, it decelerates vascular sprouting in the periphery of the retina for the superficial vascular layer and completely halts the development of the deep and intermediate vascular layers. Upon return to room air (21% oxygen, P12-P17), the avascular portion of the retina becomes ischemic triggering both intraretinal physiologic revascularization and epiretinal pathologic neovascularization processes (ROP Phase II) [2, 40, 41]. The mouse OIR model lends itself to studies investigating the angiogenic and anti-angiogenic properties of various factors and treatment modalities.

Although the expression of Bdnf mRNA in OIR-P12 retina was comparable with that of room air-P12, the mRNA expression of Bdnf was downregulated in retinas from OIR-P15 and remained low in OIR-P17 retinas compared with room-air-P15 and room air-P17 retinas. Similarly, Hellgren and colleages reported similar downregulation of Bdnf mRNA expression in retinas from mice exposed to OIR conditions as compared with retinas from mice reared in room air [26]. This is consistent with the lower level of BDNF detected in the serum from premature babies with ROP compared with premature babies who do not develop ROP [25, 26]. Although we noted a decrease in BDNF mRNA levels in OIR-P15 samples compared with room air-P15, we did not determine whether these changes in mRNA level translates to changes in BDNF protein levels. We did not detect a significant increase in Bdnf mRNA levels in OIR-P12 retinas compared with room air-P12 retina. Thus, indicating that exposure to hyperoxia minimally affected the Bdnf mRNA levels. This in contrast to the reported decrease in mRNA and protein levels of BDNF in the carotid body exposure to hyperoxia for 7 days [42]. In addition, the expression of BDNF in peribranchial smooth muscle of neonatal rats was shown to increase after exposure to high oxygen levels [43]. Cheng et al also recently reported that the level of BDNF in the retina increased after exposure to OIR conditions [44], which is in contrast to our observation here and those reported by other groups [25, 26]. The causes underlying these differences remains unclear and need to be further studied.

The expression data of the Ntrk2 gene showed the level of TrkB.T1 variant to be predominant as compared with TrkB.FL variant. While TrkB.T1 variant is still an important Bdnf receptor, it lacks the catalytic tyrosine kinase domain, which makes it behave as a dominant-negative receptor by binding excess Bdnf, which could impede Bdnf-Trkb.FL signaling [36]. This may explain, in part, the lack of DHF protective effects against vessel obliteration and neovascularization in retinas from OIR mice. Furthermore, DHF treatment resulted in reduced phosphorylation levels of TrkB protein during OIR. This could similarly contribute to the noted lack of protective effects against retinal vascular damage during OIR.

Lack of DHF protective effect against hyperoxia-induced vessel obliteration and ischemia-mediated neovascularization during OIR could be attributed to several factors which deserve further investigation in future experiments. Hypoxia-induced transcription factors (HIFs) mediate cellular responses to varying oxygen levels by inducing the expression of genes critical for vascular development and homeostasis, such as VEGF [45–47]. After exposure to high oxygen levels, the HIFs are degraded, and VEGF expression is downregulated. Reduced VEGF expression can block DHF activity by inhibiting the phosphorylation of TrkB protein. Consistent with this notion, Segatto et al. reported that VEGF neutralization by anti-VEGF specific antibodies inhibited the phosphorylation of TrkB protein, inducing apoptosis and autophagy in rabbit retina [48]. Degradation of HIFs after exposure to hyperoxia results in lower total protein level of TrkB [49], reducing the ability of DHF to exert a protective effect. DHF also binds and inhibits the activity of VEGFR2, beside its effect on TrkB in rat retina [50]. Such inhibitory effect on VEGFR2 may compromise its activity on TrkB protein and result in the lack of vascular protection of DHF as we observed here in the mouse retina after exposure to hyperoxia.

Our data here showing lack of a protective impact of DHF during OIR is consistent with conflicting reports in the literature. Although DHF is reported to work as BDNF mimic by activating TrkB and when used, it has neuroprotective effects on relevant neurological diseases [20–22], others reported that they were not able to detect DHF ability to bind and activate TrkB protein in in vitro settings [51, 52]. Furthermore, Zhou et al. reported that DHF fails to inhibit the development of Alzheimer-associated phenotypes [53]. Thus, DHF function may have a narrow therapeutic range or be limited to certain disease states. In addition, given the tendency of DHF to scavenge intracellular reactive oxygen species may limit its TrkB activity under oxidative stress conditions such as OIR [54, 55].

In conclusion, to the best of our knowledge, this is the first report that examines the protective effect of DHF on retinal vasculature integrity during OIR. Our findings showed that treating animals with DHF does not provide significant protection against hyperoxia-induced vascular obliteration and ischemia-mediated neovascularization and revascularization. Plausible reasons for these observations are that high oxygen level could result, directly and/or indirectly, in decreased total TrkB protein levels, its activation and signaling capacity that are subject of future investigation. Thus, our studies suggest that DHF does not have the same kind of protective effect on the retinal vasculature as reported on neurons.

## Supporting information

S1 File (PDF)
