# Supplementary material for: 7, 8-Dihydroxyflavone, a TrkB receptor agonist, provides minimal protection against retinal vascular damage during oxygen-induced ischemic retinopathy
Source: PLoS One. 2021 Dec 2;16(12):e0260793. doi: 10.1371/journal.pone.0260793 (PMC8638941; doi:10.1371/journal.pone.0260793)
Supplement: S1 File — (PDF) [file pone.0260793.s001.pdf]

Retinas from Room air B6j mice injected with DHF WB #1

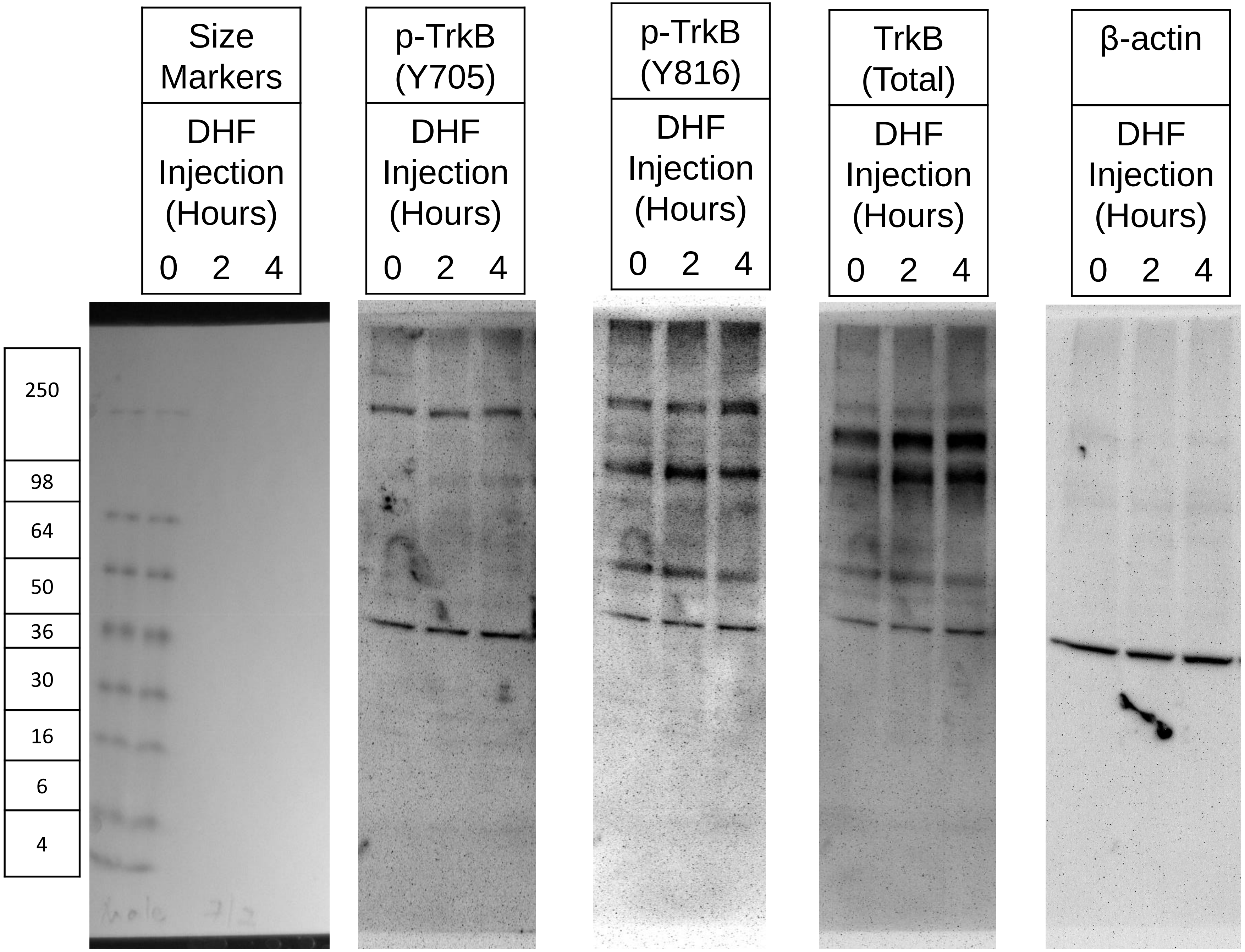

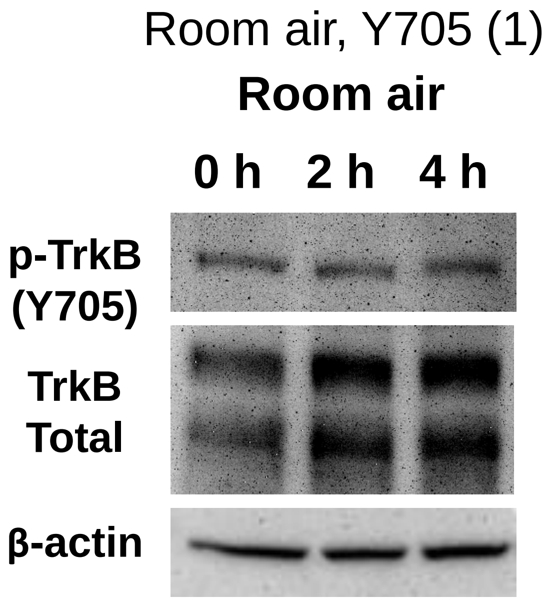

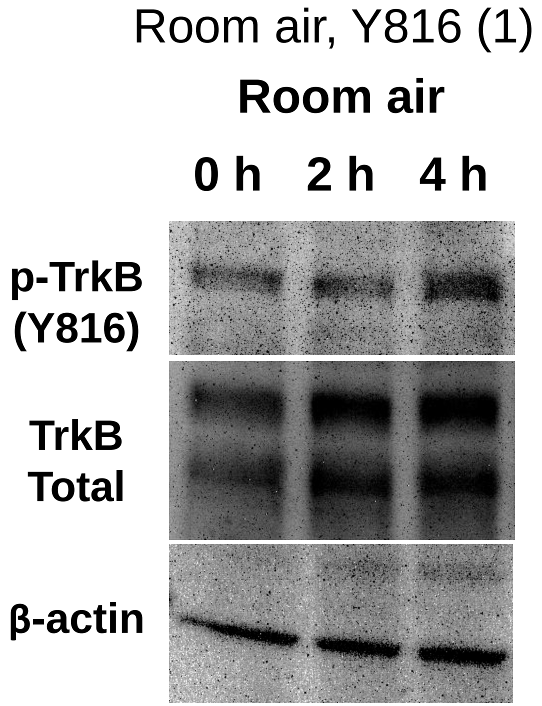

Retinas from Room air B6j mice injected with DHF WB #2

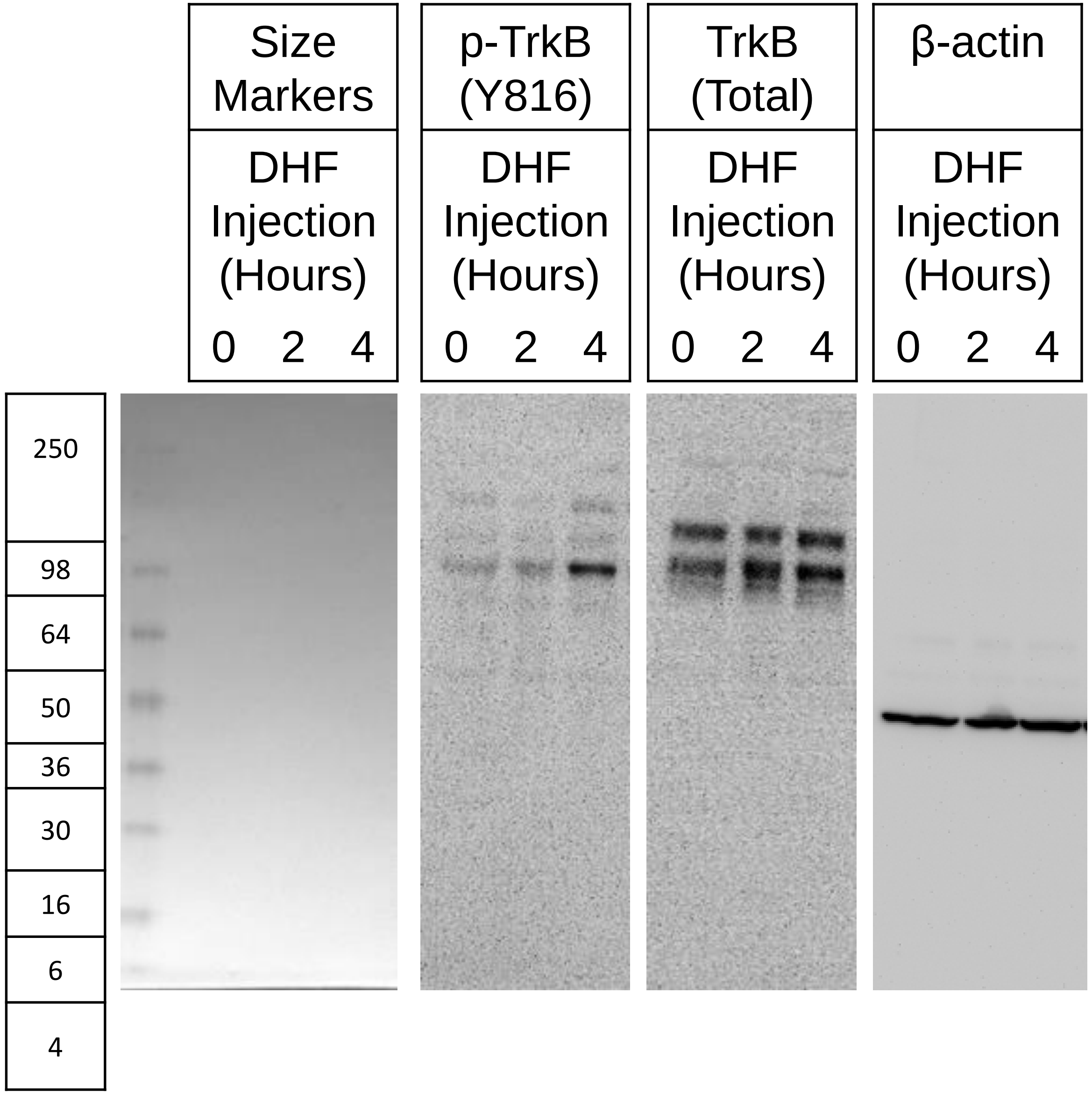

Room air, Y816 (2)

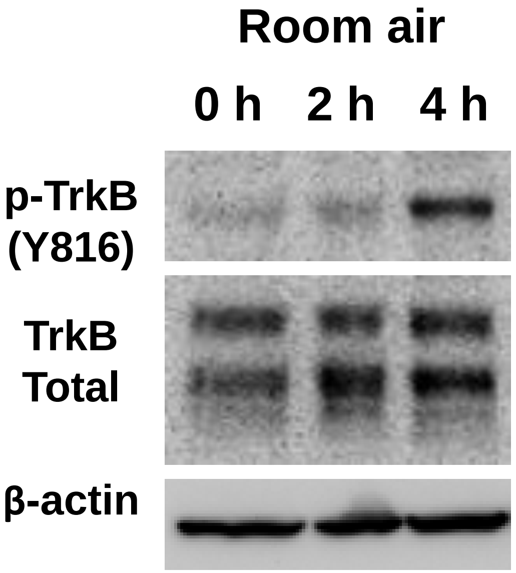

Retinas from Room air B6j mice injected with DHF WB #3

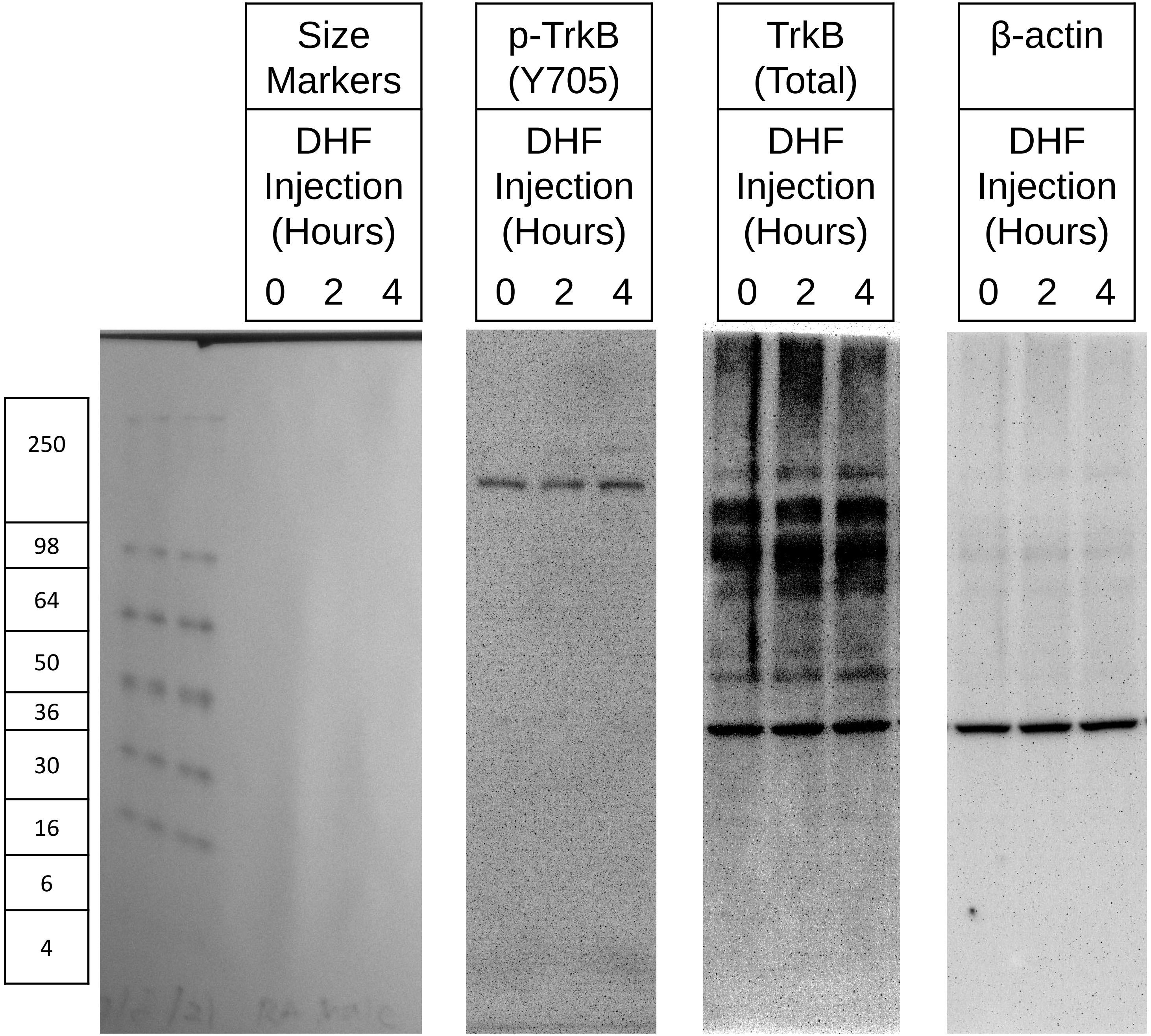

Room air, Y705 (2)

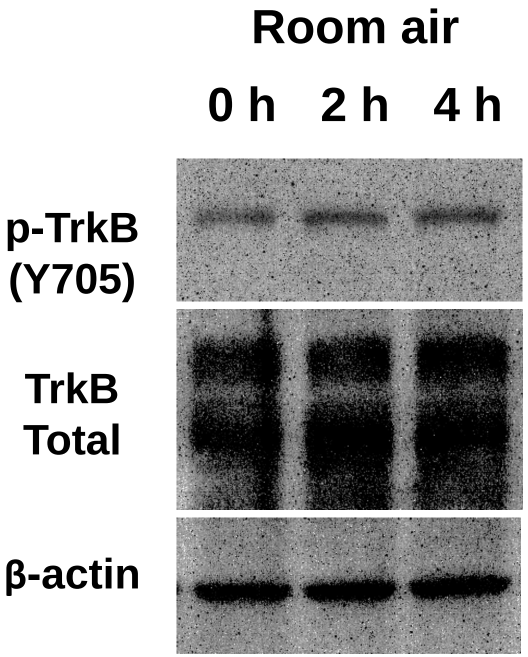

Room air, Y705 (1)

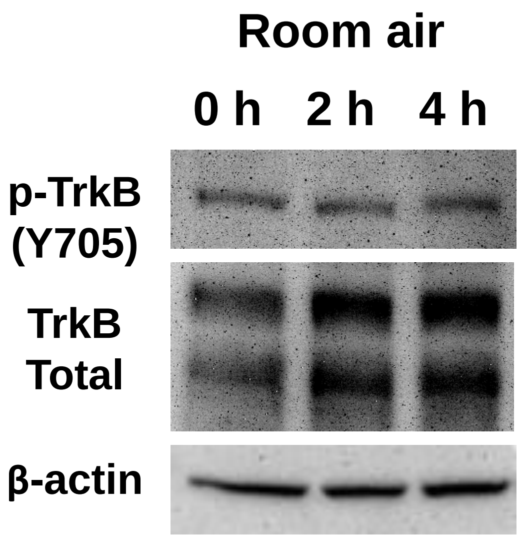

Room air, Y705 (2)

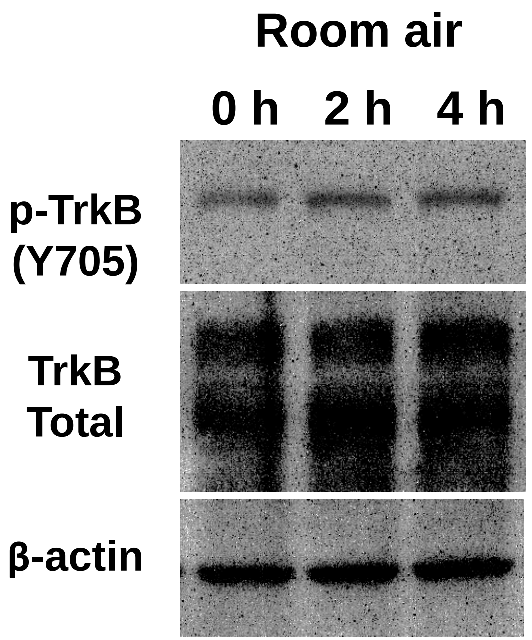

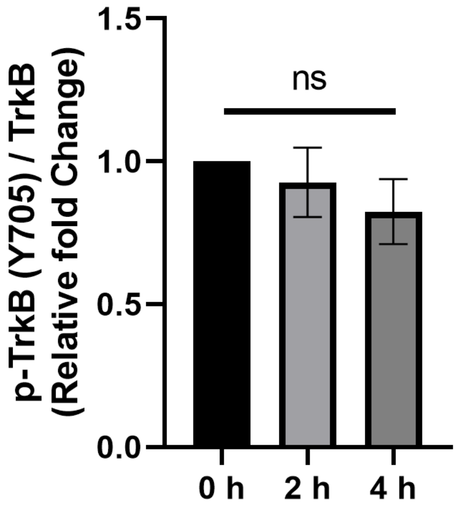

Rooma air Y705 (1)				
Sample	p-TrkB Y705	TrkB Total	p-TrkB / TrkB	Nomalized by 0 h
	Band size	Band size		
0 h	19950.856	34103.94	0.585001	1
2 h	16574.827	33704.563	0.491768	0.84062698
4 h	14816.291	34027.593	0.43542	0.74430561

Room air Y705 (2)				
Sample	p-TrkB Y705	TrkB Total	p-TrkB / TrkB	Nomalized by 0 h
	Band size	Band size		
0 h	16813.46	37189.92	0.452097	1
2 h	16635.01	36341.47	0.457742	1.012484741
4 h	18867.94	46105.1	0.409238	0.905197715

	0 h	2 h	4 h
Y705 (1)	1	0.840627	0.744306
Y705 (2)	1	1.012485	0.905198

Dunnett's multiple comparisons test	Mean Diff.	95.00% CI of diff.	Significant?	Summary	Adjusted P Value	A-?	
0 h vs. 2 h	0.07344	-0.2982 to 0.4451	No	ns	0.6967	B	2 h
0 h vs. 4 h	0.1752	-0.1964 to 0.5469	No	ns	0.2592	C	4 h

Room air, Y816 (1)

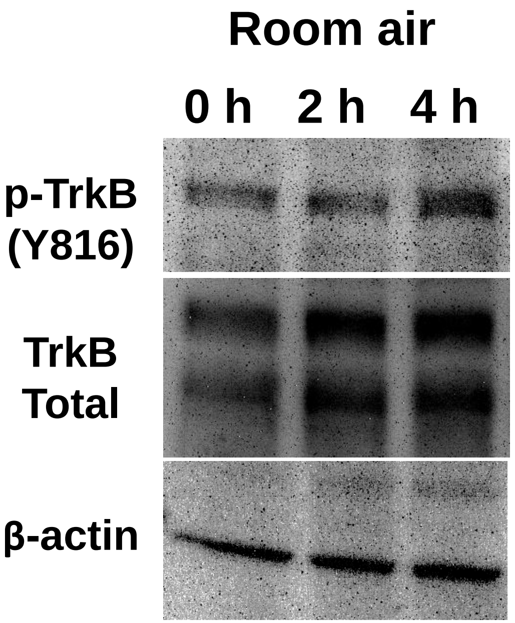

Room air, Y816 (2)

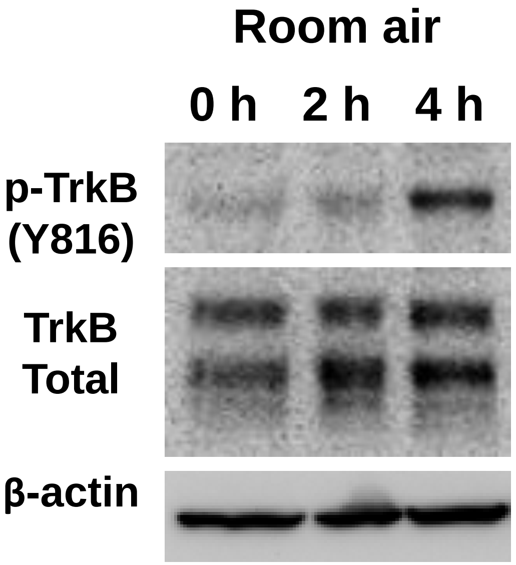

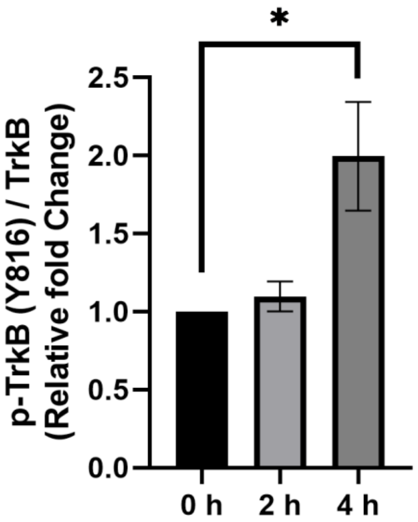

Room air Y816 (1)				
Sample	p-TrkB Y816	TrkB Total	p-TrkB / TrkB	Nomalized by 0 h
	Band size	Band size		
0 h	20220.42	35536.41	0.569006	1
2 h	23831.1	40674.39	0.585899	1.029689765
4 h	39754.25	39932.44	0.995538	1.749609346

Room air Y816 (2)				
Sample	p-TrkB Y816	TrkB Total	p-TrkB / TrkB	Nomalized by 0 h
	Band size	Band size		
0 h	21426.765	45728.978	0.46856	1
2 h	20097.794	36806.279	0.546043	1.16536338
4 h	47491.643	45232.401	1.049947	2.24079663

	0 h	2 h	4 h
Y816 (1)	1	1.02969	1.749609
Y816 (2)	1	1.165363	2.240797

Dunnett's multiple comparisons test	Mean Diff.	95.00% CI of diff.	Significant?	Summary	Adjusted P Value	A-?	
0 h vs. 2 h	-0.09753	-0.9019 to 0.7069	No	ns	0.8606	B	2 h
0 h vs. 4 h	-0.9952	-1.800 to -0.1908	Yes	*	0.0285	C	4 h

Retinas from OIR B6j mice injected with DHF WB #1

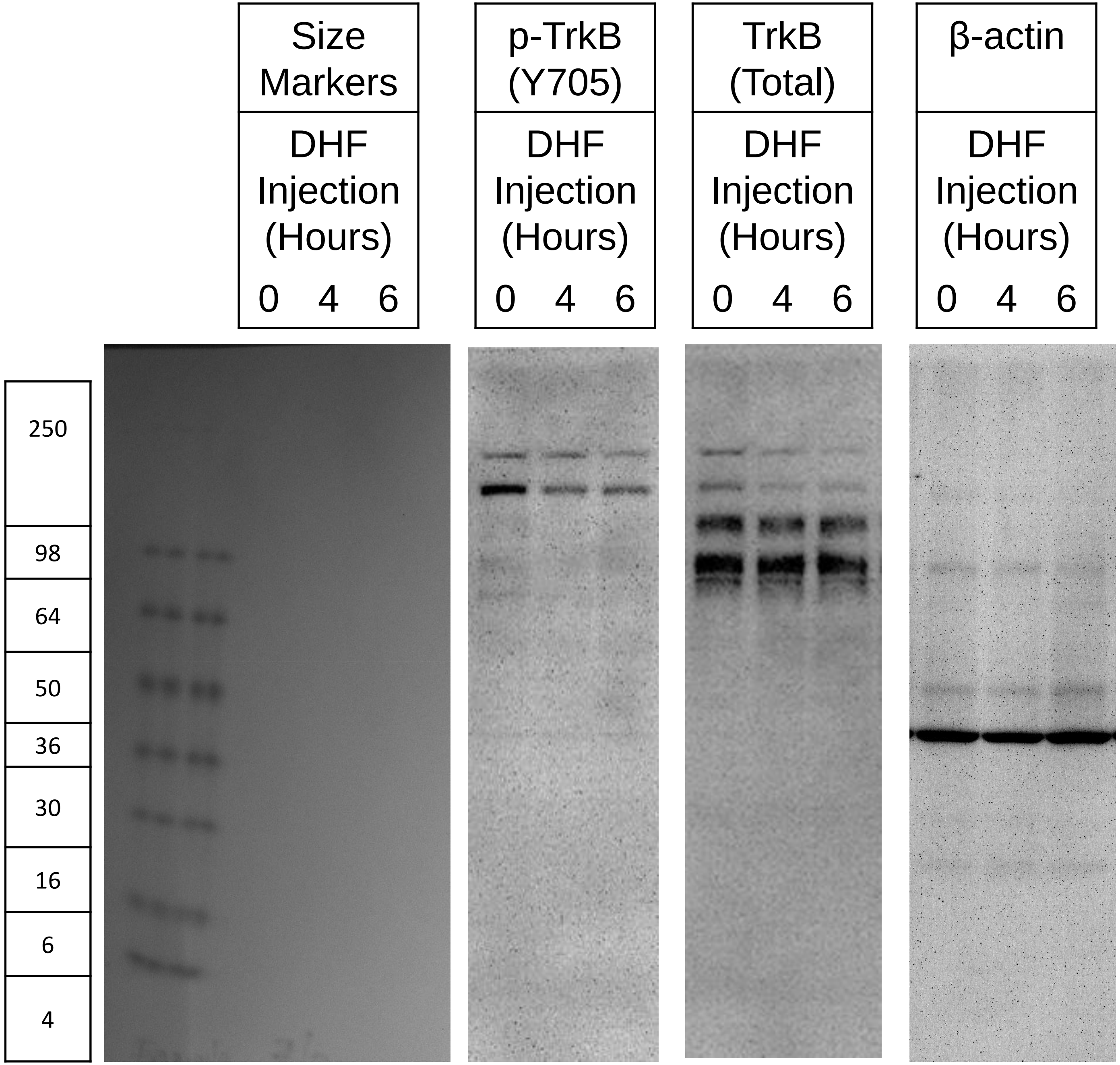

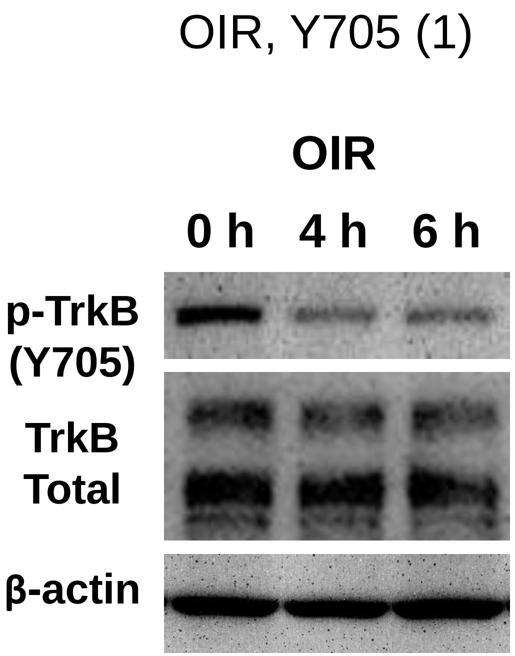

Retinas from OIR B6j mice injected with DHF WB #2

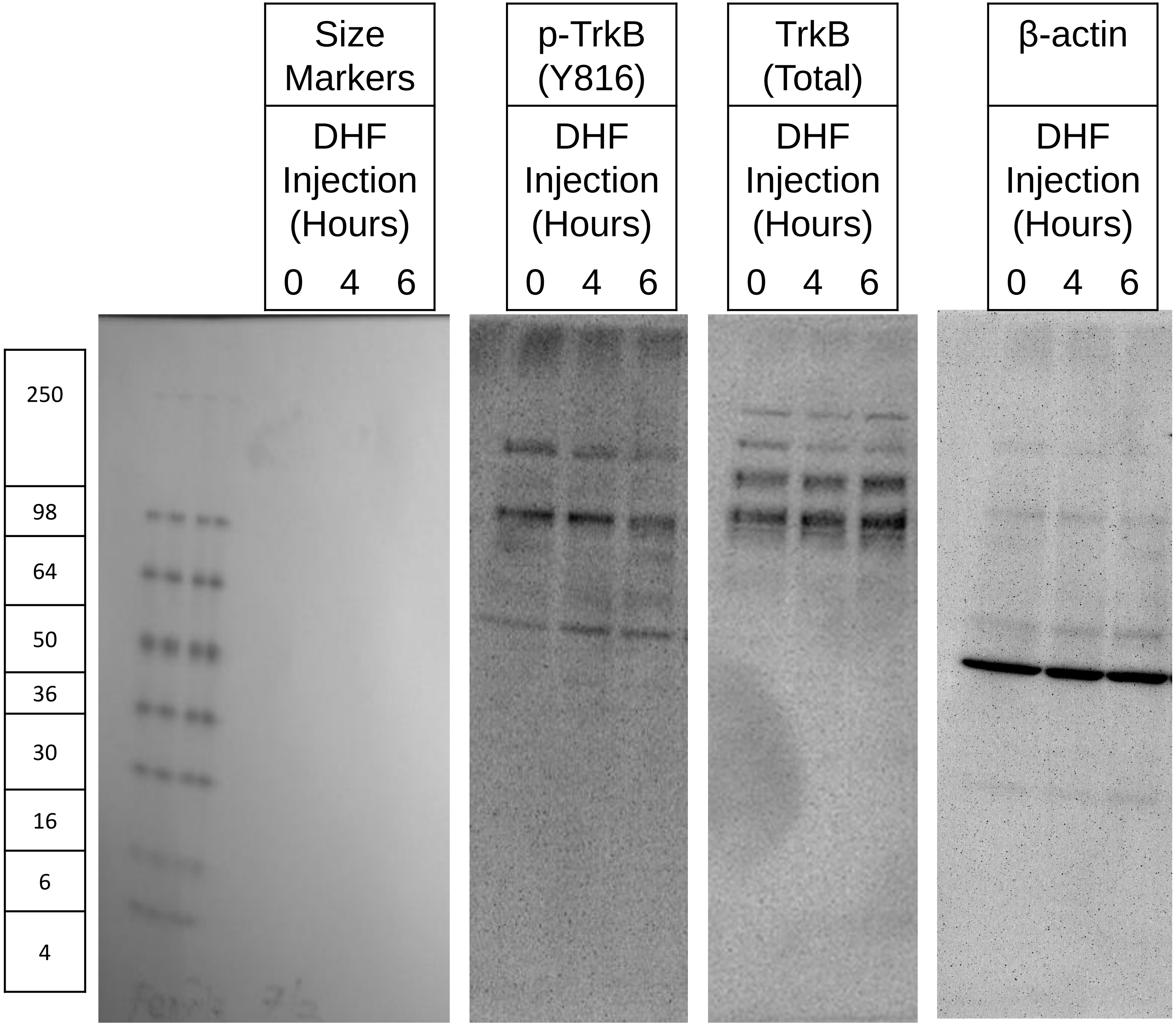

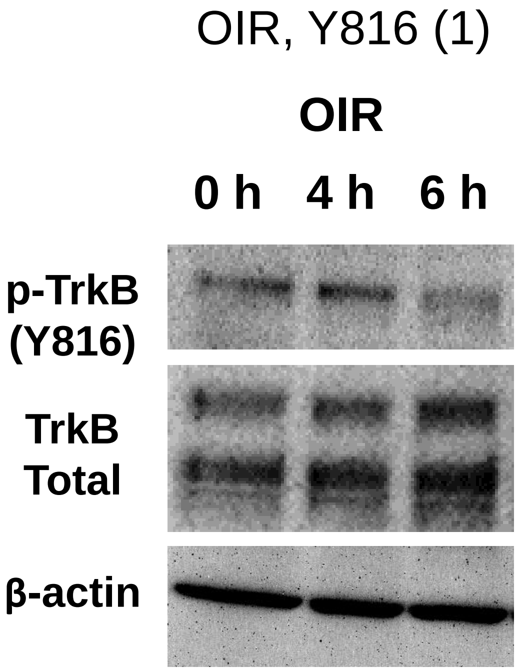

Retinas from OIR B6j mice injected with DHF WB #3

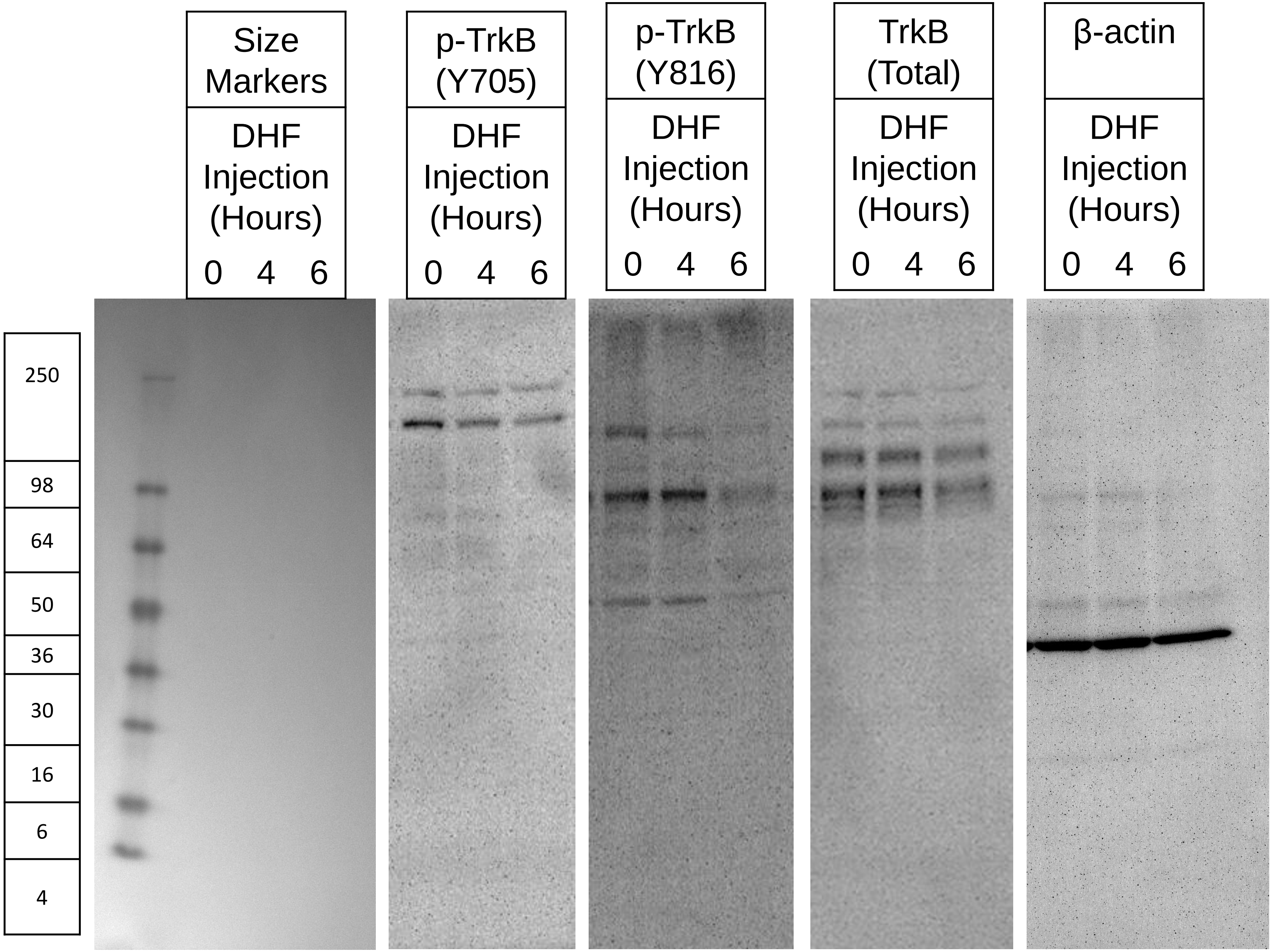

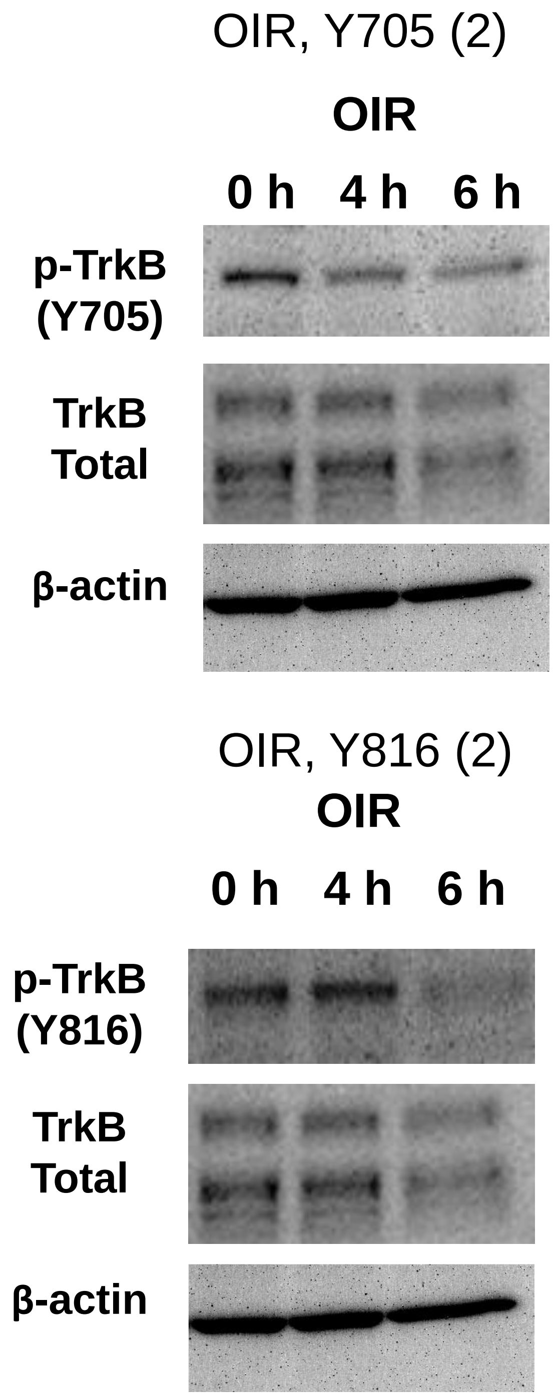

OIR, Y705 (1)

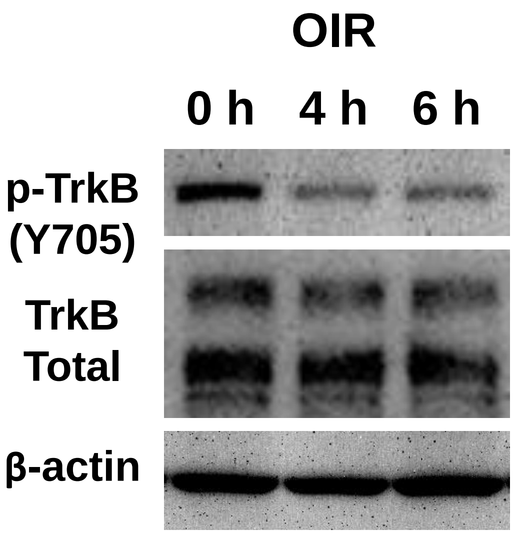

OIR, Y705 (2)

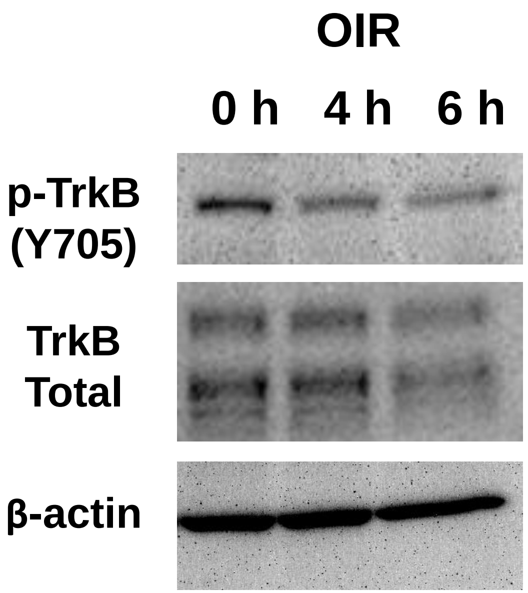

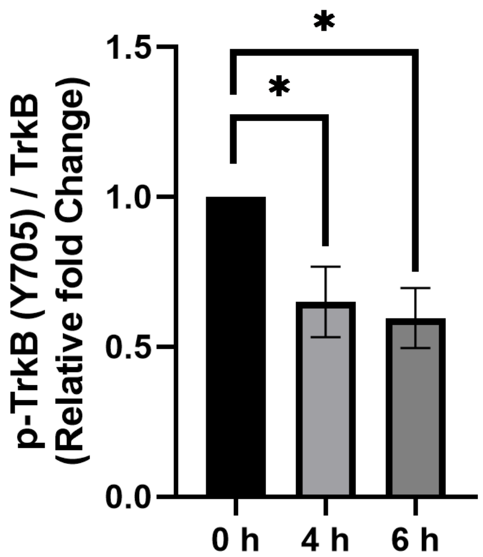

OIR Y705 (1)				
Sample	p-TrkB Y705	TrkB Total	p-TrkB / TrkB	Nomalized by 0 h
	Band size	Band size		
0 h	51351.66	49665.59	1.033948	1
4 h	22970.3	39213.2	0.58578	0.566546
6 h	20939.42	38554.13	0.543117	0.525285

OIR Y705 (2)				
Sample	p-TrkB Y705	TrkB Total	p-TrkB / TrkB	Nomalized by 0 h
	Band size	Band size		
0 h	46799.3	46531.4	1.005757	1
4 h	33366.51	45268.66	0.737077	0.732858
6 h	27378.15	40877.86	0.669755	0.665921

	0 h	4 h	6 h
Y705 (1)	1	0.566546	0.525285
Y707 (2)	1	0.732858	0.665921

Dunnett's multiple comparisons test	Mean Diff.	95.00% CI of diff.	Significant?	Summary	Adjusted P Value	A-?	
0 h vs. 4 h	0.3503	0.006482 to 0.6941	Yes	*	0.0477	B	4 h
0 h vs. 6 h	0.4044	0.06058 to 0.7482	Yes	*	0.0327	C	6 h

OIR, Y816 (1)

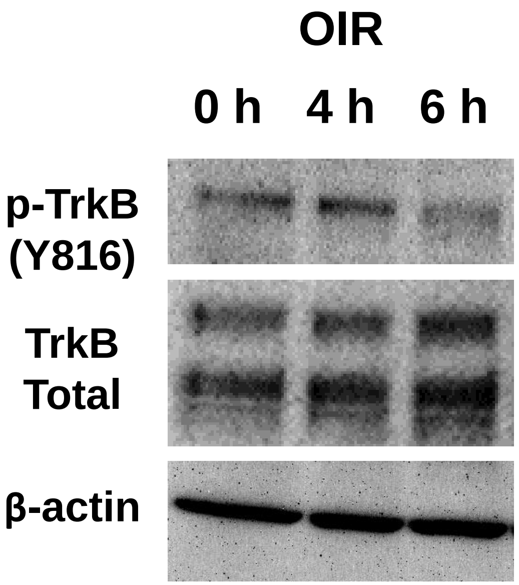

OIR, Y816 (2)

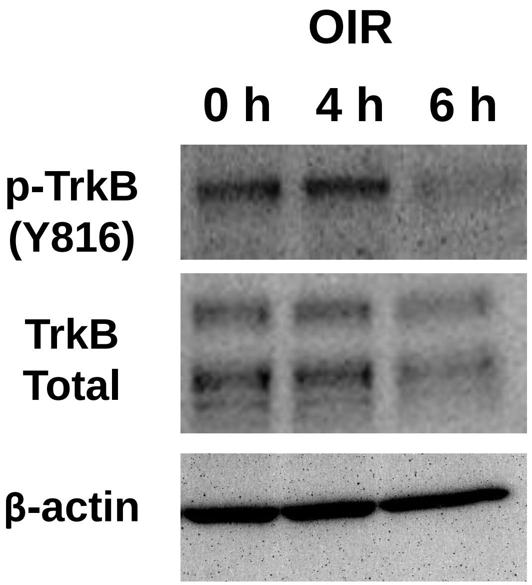

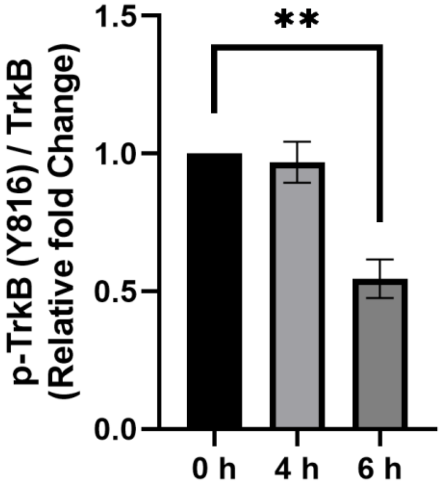

OIR Y816 (1)				
Sample	p-TrkB Y816	TrkB Total	p-TrkB / TrkB	Nomalized by 0 h
	Band size	Band size		
0 h	57395.48	37230.25	1.541636	1
4 h	43209.2	30590.05	1.412525	0.916251
6 h	32417.77	35316.91	0.917911	0.595413

OIR Y816 (2)				
Sample	p-TrkB Y816	TrkB Total	p-TrkB / TrkB	Nomalized by 0 h
	Band size	Band size		
0 h	47237.44	46531.4	1.015173	1
4 h	46922.86	45268.66	1.036542	1.021049
6 h	20569.03	40877.86	0.503183	0.495662

	0 h	4 h	6 h
Y816 (1)	1	0.916251	0.595413
Y816 (2)	1	1.021049	0.495662

Dunnett's multiple comparisons test	Mean Diff.	95.00% CI of diff.	Significant?	Summary	Adjusted P Value	A-?	
0 h vs. 4 h	0.03135	-0.1970 to 0.2597	No	ns	0.8279	B	4 h
0 h vs. 6 h	0.4545	0.2261 to 0.6829	Yes	**	0.0076	C	6 h
